# Topical NSAIDs for chronic musculoskeletal pain: systematic review and meta-analysis

**DOI:** 10.1186/1471-2474-5-28

**Published:** 2004-08-19

**Authors:** Lorna Mason, R Andrew Moore, Jayne E Edwards, Sheena Derry, Henry J McQuay

**Affiliations:** 1 Pain Research and Nuffield Department of Anaesthetics, University of Oxford, Oxford Radcliffe Hospital, The Churchill, Headington, Oxford, OX3 7LJ, UK

## Abstract

A previous systematic review reported that topical NSAIDs were effective in relieving pain in chronic conditions like osteoarthritis and tendinitis. More trials, a better understanding of trial quality and bias, and a reclassification of certain drugs necessitate a new review.

Studies were identified by searching electronic databases, and writing to manufacturers. We identified randomised, double blind trials comparing topical NSAID with either placebo or another active treatment, in adults with chronic pain. The primary outcome was a reduction in pain of approximately 50% at two weeks, and secondary outcomes were local and systemic adverse events and adverse event-related withdrawals. Relative benefit and number-needed-to-treat (NNT), and relative harm and number-needed-to-harm (NNH) were calculated, and the effects of trial quality, validity and size, outcome reported, and condition treated, were examined by sensitivity analyses.

Twelve new trials were added to 13 trials from a previous review. Fourteen double blind placebo-controlled trials had information from almost 1,500 patients. Topical NSAID was significantly better than placebo with relative benefit 1.9 (95% confidence interval 1.7 to 2.2), NNT 4.6 (95% confidence interval 3.8 to 5.9). Results were not affected by trial quality, validity or size, outcome reported, or condition treated. Three trials with 764 patients comparing a topical with an oral NSAID found no difference in efficacy. Local adverse events (6%), systemic adverse events (3%), or the numbers withdrawing due to an adverse event were the same for topical NSAID and placebo.

Topical NSAIDs were effective and safe in treating chronic musculoskeletal conditions for two weeks. Larger and longer trials are necessary to fully elucidate the place of topical NSAIDs in clinical practice.

## Background

A systematic review of topical NSAIDs reported that they were effective for relieving pain in both acute and chronic conditions [1]. Number-needed-to-treat (NNT), the number of patients that need to be treated for one to benefit from a particular drug, who would not have benefited from placebo, was used to estimate efficacy. In chronic conditions, NNT for topical NSAIDs at two weeks was 3.1 (2.7 to 3.8).

There are three reasons why an updated review of topical NSAIDs in chronic pain is needed. First, we have a better appreciation of factors that can introduce bias [2-4], and would not now accept trials that were not double blind, or were very small. Second, topical salicylate and benzydamine are no longer classed as topical NSAIDs [5]. Thirdly, there are now more trials. We believed that updating the review would improve efficacy estimates for topical NSAIDs, with a prior intent to determine efficacy for individual drugs.

## Methods

### Searching

Relevant studies were sought regardless of publication language, type, date or status. Studies included in the previous review were considered for inclusion, and the Cochrane Library, MEDLINE and PreMedline, EMBASE and PubMed, were searched for relevant studies published since the last review, for the years 1996 to April 2003. The search strategy included "application: topical" together with "cream", "gel" etc, together with generic names of NSAIDs, and proprietary preparations of topical treatment in which the principal active ingredient was an NSAID [6,7] (additional file 1:search strategy). Reference lists of retrieved articles were also searched. We wrote to 20 pharmaceutical companies in the UK, 66 in continental Europe, and two in North America, known to manufacture topical NSAIDs, asking if they could supply papers.

### Selection

We identified reports of randomised, double blind, active or placebo-controlled trials in which treatments were given to adult patients with moderate to severe chronic pain resulting from musculoskeletal or other painful disorders. We excluded treatments for mouth or eye diseases. At least ten patients had to be randomised to a treatment group and application of treatment had to be at least once daily. Outcomes closest to two weeks (but at least seven days) were extracted. Longer outcomes were also accepted when available.

### Quality and validity assessment

Trial quality was assessed using a validated three-item scale with a maximum quality score of five [8]. Included studies had to score at least two points, one for randomisation and one for blinding. A sixteen-point scale was used to assess trial validity [9]. Quality and validity assessments were made independently by at least two reviewers and verified by one other reviewer. Disputes were settled by discussion between all reviewers.

### Outcomes

We defined our own outcome of clinical success, representing approximately a 50% reduction in pain [1]. This was either the number of patients with a "good" or "excellent" global assessment of treatment, or "none" or "slight" pain on rest or movement (or comparable wording) measured on a categorical scale. A hierarchy of outcomes was used to extract efficacy information [1], shown below in order of preference:

1) number of patients with a 50% or more reduction in pain

2) patient reported global assessment of treatment

3) pain on movement

4) pain on rest or spontaneous pain

5) physician or investigator global assessment of treatment

In addition, the number of patients showing undefined "improvement" was also accepted. All of these outcomes were grouped together as a "success", and categories 1–4 were used as preferred outcomes in the sensitivity analysis.

Secondary outcomes were extracted from included papers that reported them. These were the number of patients (i) reporting one or more local adverse event (itching, stinging, rash), (ii) reporting one or more systemic adverse event (iii) withdrawing from trials due to adverse events.

### Quantitative data synthesis

The number of patients randomised into each treatment group (intention to treat) was used in the efficacy analysis. Information was pooled for the number of patients in each trial approximating at least 50% pain relief, or similar measure, for both topical NSAID and control. These were used to calculate NNT with a 95% confidence interval (CI) [10]. Relative benefit and relative risk estimates with 95% CIs were calculated using the fixed effects model [11]. A statistically significant benefit of topical NSAID over control was assumed when the lower limit of the 95% confidence interval (CI) of the relative benefit was greater than one. A statistically significant benefit of control over active treatment was assumed when the upper limit of the 95% CI was less than one. Homogeneity of trials was assessed visually [12-14]. Number-needed-to-harm (NNH) and relative risk were calculated in the same way as for NNTs and relative benefit. All calculations were performed using Microsoft Excel X for the Macintosh and RevMan 4.2. In sensitivity analyses the z test was used [15]. QUOROM guidelines were followed [16].

### Sensitivity analysis

Our prior intention was to perform sensitivity analyses on pooled outcomes using the z test [15] for quality score (2 versus 3 or more), validity score (8 or less versus 9 or more), trial size (less than 40 patients per group versus more than 40 patients per group), reported outcome (higher versus lower preference), drug, and condition treated (knee osteoarthritis versus other musculoskeletal). At least three studies had to be available in each category before information was pooled.

## Results

### Study characteristics

Ten out of the 20 UK companies, and two out of the 66 continental European companies replied to our request for studies. Only three companies supplied useful material, either published studies or bibliographies. None provided unpublished material.

Searches identified 60 target papers, but 35 were excluded; 23 studies failed to meet the inclusion criteria and 12 had no useable data. Twenty-four of these 60 target papers were included in the previous review. We included 13 of those in this review, and excluded 11; seven were not double blind, two compared a salicylate with placebo or oral analgesics, one did not have daily application, and one had insufficient data (additional file 2: excluded studies, additional file 3: QUOROM flow diagram).

Twenty-five trials therefore met the selection criteria, 12 of which were additional trials. Fifteen trials had only placebo controls [17-31], seven only active controls [32-38], and three had both placebo and active controls [39-41]. Of the 10 active controlled trials, four compared a topical NSAID with a different topical NSAID, three compared a topical NSAID with a different oral NSAID, and one each compared a topical NSAID with a homeopathic gel, a topical rubefacient, and topical trinitroglycerin (GTN). Details of all included studies with outcomes and quality and validity scores are in additional files 4 (Outcome details of placebo-controlled trials) and additional files 5 (Outcome details of active-controlled trials).

Patients were generally over 40 years old, predominantly with musculoskeletal disorders, and with baseline pain of moderate to severe intensity. Fourteen studies examined general musculoskeletal conditions, and eleven examined osteoarthritis (9 studies of the knee, one of finger joints, and one of mixed sites). Five studies in osteoarthritis specified use of a standard scale (ACR, Kellgren and Lawrence, ISK) to assess the severity of disease, four specified that the disease was radiologically confirmed, one specified that patients had "well documented mild osteoarthritis", and one made no statement.

Quality scores were high, with 16/18 placebo controlled and 9/10 active controlled trials scoring 3 or more points out of a maximum of 5. Validity scores were also high, with 14/18 placebo controlled and 8/10 active controlled trials scoring 9 or more out of a maximum of 16 (additional files 4 and 5).

### Placebo controlled trials

Dichotomous information was available to pool from 14 placebo controlled trials for efficacy, from 16 placebo controlled trials for local adverse events, 17 placebo controlled trials for systemic adverse events, and from 11 placebo controlled trials for adverse event related withdrawals.

#### Efficacy

**Figure 1:**
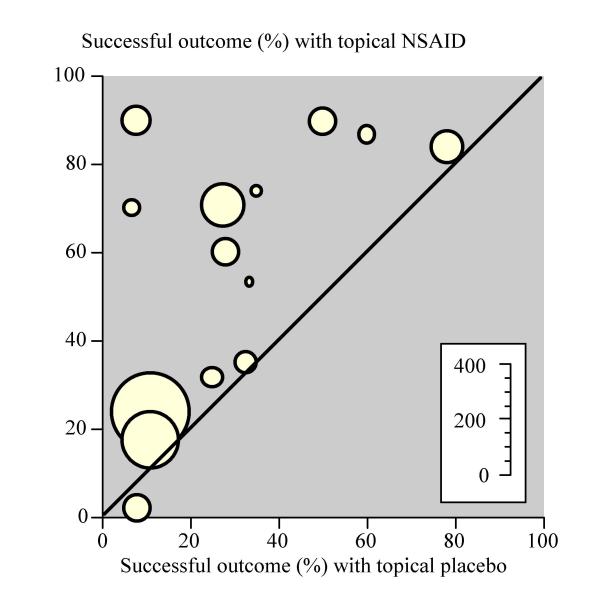
Topical NSAIDs in chronic musculoskeletal pain Randomised double-blind studies of topical NSAID compared to topical placebo for two-week outcome of successful treatment. Inset scale shows size of individual trials.

Fourteen trials (1,502 patients) provided data on efficacy. Topical NSAIDs were significantly better than placebo (Table 1). The mean placebo response rate was 26% ranging from 7% to 78%. The mean treatment response rate was 48% ranging from 2% to 90% (Figure 1). The NNT was 4.6 (95% CI 3.8 to 5.9) for one patient to experience improvement in chronic musculoskeletal pain at two weeks with topical NSAIDs, compared with placebo.

**Figure 2:**
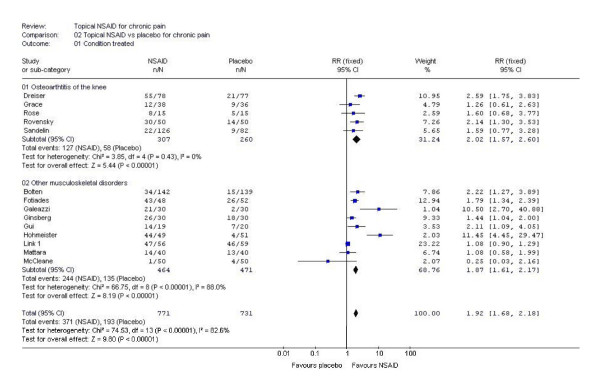
Analysis of trials of topical NSAIDs in chronic musculoskeletal pain by condition. This Forrest plot was created using RevMan 4.2. Details of the statistical tests used can be found in the Cochrane Handbook.

Sensitivity analyses (Table 1) showed no significantly greater effect for low quality trials (quality score 2/5) compared with higher quality trials (quality score 3–5/5) (z = 1.69, p = 0.091). There was no significant difference for smaller versus larger trials using 50 patients per group (median group size for topical NSAID was 49) as a cut off (z = 0.40, p = 0.69), for preferred outcomes versus lower preference outcomes (physician determined or general improvement) (z = 1.56, p = 0.12), or for patients with knee osteoarthritis compared with other musculoskeletal conditions (z = 0.99, p = 0.32) (Figure 2). The 10 trials with both a quality score of 3/5 or greater and a validity score of 9/16 or greater had an NNT of 4.4 (95% CI 3.6 to 5.6). There were insufficient data to allow comparisons of efficacy between different NSAIDs.

#### Harm

All 18 placebo controlled trials (2,032 patients) provided some information on adverse events (Table 2). There was no statistically significant difference between topical NSAID and topical placebo for the number of patients experiencing local adverse events (6%), systemic adverse events (3%), or the number withdrawing due to an adverse event (1%). With topical NSAID or topical placebo, local adverse events were usually described as rash, itching or stinging, and were predominantly mild.

### Active controlled trials

#### Efficacy

There was sufficient information to pool results only from the three trials comparing a topical NSAID with an oral NSAID in patients with osteoarthritis of the knee or finger joints. One trial [34] compared piroxicam 0.5% gel with oral ibuprofen 1200 mg daily, another [38] compared diclofenac 1% gel with oral ibuprofen 1200 mg daily, and the third [41] compared eltenac 1% gel with oral diclofenac 100 mg daily. In these trials, with 764 patients, 37% had a successful outcome both with topical NSAID and oral NSAID. There was no statistically significant difference (relative risk 1.1; 95% CI 0.9 to 1.3). The other seven studies used different topical preparations and different comparators in small trials (additional file 5: Outcome details of active-controlled trials).

#### Harm

Eight of the active controlled trials (1,461 patients) provided some information on adverse events (Table 2). In two active controlled trials comparing topical with oral NSAID, local adverse events occurred more frequently (8%) with topical than with oral NSAID (3%). Systemic adverse events and adverse event withdrawals did not differ between topical and oral NSAID. No study documented specific instances of upper gastrointestinal bleeding or symptomatic ulcers.

## Discussion

Patients in these trials all had moderate to severe baseline pain, and for those with osteoarthritis, disease severity was generally mild to moderate. Patients with most severe disease were specifically excluded in several trials because authors regarded topical NSAID to be inappropriate for their treatment.

Both the original and this updated review concluded that topical NSAIDs were effective in chronic conditions. However, removing trials of lower quality, and topical agents that are not now regarded as topical NSAIDs, increased (worsened) the NNT from 3.1 (95% CI 2.7 to 3.8) to 4.7 (95% CI 3.8 to 5.9) for the outcome of at least half pain relief at two weeks for all topical NSAIDs compared to placebo. For every four or five patients with chronic pain treated with topical NSAID, one would benefit who would not have done with placebo. Three trials comparing topical with oral NSAID found no difference in efficacy.

There are a number of aspects of this review that might question this demonstration of efficacy. The trials spanned several decades and retrospective examination finds fault with them in several respects. Many trials were small, and small size can allow chance effects to influence treatment and placebo event rates [4]. Different preparations were used, with different formulations, concentrations of active ingredient, and application schedules. Reported outcomes were not consistent, and a hierarchy of outcomes had to be constructed. It was inevitable that there would be some clinical heterogeneity, even when similar patients were treated, and when trials were both randomised and double blind, and of appropriate duration.

We addressed these limitations with pre-planned sensitivity analyses. Using studies with higher quality and validity scores, larger size, or higher rather than lower preference outcomes made no difference. Patients treated for knee osteoarthritis derived the same degree of pain relief as those treated for general musculoskeletal conditions. The evidence was that topical NSAIDs were effective whatever strategy was used for sensitivity analysis, improving the robustness of the overall result.

A possible criticism might be that there has been selective publication of trials showing topical NSAIDs to be effective, and suppression of trials where there was no difference between topical NSAID and placebo. Funnel plots do not reliably detect publication bias [13,14], so we did not use them or make any adjustment for possible publication bias [42]. We did approach every company in the world that we could identify as being involved with topical NSAID manufacture or sale for any additional unpublished trials, but no more unpublished material was identified. When unpublished material is found, it often does not change the relevance of a result [43-45].

It is important to emphasise that both active and placebo treatments were rubbed on, making any effect of rubbing equal in both groups. The mean placebo response in the included trials was 26%, compared with the mean response of 48% with topical NSAID. The response with placebo is consistent with that found in acute and chronic pain with a variety of conditions and endpoints [46].

Local adverse events were reported with equal frequency for topical NSAID and topical placebo in placebo-controlled trials, but more often for topical NSAID than oral NSAID in active controlled trials. There were no differences between topical NSAID and topical placebo, or topical NSAID and oral NSAID, for systemic adverse events, or withdrawals due to adverse events. Studies of short duration will not capture important long-term safety information, and this may be important for ongoing applications of gels, creams or sprays in chronic conditions. There is, however, information that indicates that topical NSAIDs do not cause the gastrointestinal harm found with oral NSAIDs [47], nor are they associated with increased renal failure [48].

Clearly there is a body of evidence to support the efficacy of topical NSAIDs in chronic painful musculoskeletal conditions. Despite removing smaller studies that were not double blind, and substituting newer, larger trials of higher quality, topical NSAIDs remained effective, though the NNT was higher (worse) than originally estimated [1]. More information of high quality is required, to compare the relative efficacy of topical and oral NSAIDs, and between different topical NSAIDs.

We are able to compare the evidence for different topical analgesics in chronic musculoskeletal pain (Table 3). Systematic reviews of topical salicylate [49] and capsaicin [50], tell us what is known about those treatments. As Table 3 shows, topical NSAIDs have been tested in many more studies, and in four times as many patients as these other topical analgesics, and have the lowest (best) NNT. The limitation of this comparison is essentially the same limitation as with all these reviews, that the included trials were too short and too small to be sure of the result. Topical NSAIDs have the best evidence for chronic musculoskeletal pain, supporting the excellent evidence available in acute painful conditions [51].

## Authors' contributions

LM was involved with planning the study, searching, data extraction, analysis, and preparing a manuscript; RAM with planning, data extraction, analysis and writing the manuscript; JE with searching, data extraction, and writing; SD with data extraction, analysis, and writing; HJM with planning, analysis and writing. All authors read and approved the final manuscript.

## Competing interests

RAM & HJM have consulted for various pharmaceutical companies. RAM, HJM & JE have received lecture fees from pharmaceutical companies related to analgesics and other healthcare interventions. All authors have received research support from charities, government and industry sources at various times, but no such support was received for this work. No author has any direct stock holding in any pharmaceutical company.

## Pre-publication history

The pre-publication history for this paper can be accessed here:



## Supplementary Material

Additional File 1 Search strategy for RCTs of topical NSAIDs in chronic pain Click here for file

Additional File 2 Excluded studies Click here for file

Additional File 3 QUOROM flow diagram Click here for file

Additional File 4 Outcome details of placebo-controlled trials Click here for file

Additional File 5 Outcome details of active-controlled trials Click here for file

## Figures and Tables

**Table 1:** Summary data and sensitivity analyses for placebo controlled trials

	**Number of**		**Success/total**			
			
**Trial characteristic**	**Trials**	**Patients**	**Treatment**	**Placebo**	**RB (95% CI)**	**NNT (95% CI)**
**All trials**	14	1502	371/771	193/731	1.9 (1.7 to 2.2)	4.6 (3.8 to 5.9)
**Quality and validity**						
Quality score 3 to 5	11	1312	294/678	144/634	2.0 (1.7 to 2.4)	4.8 (3.9 to 6.4)
Quality score 2	3	190	77/93	49/97	1.6 (1.3 to 2.0)	3.1 (2.2 to 5.1)
Validity score ≥ 9 and quality score ≥ 3	10	1197	247/622	98/575	2.4 (2.0 to 3.0)	4.4 (3.6 to 5.6)
**Trial group size**						
≤ 50 patients	6	343	95/172	54/171	1.8 (1.4 to 2.3)	4.2 (3.0 to 7.4)
> 50 patients	8	1159	276/599	139/560	2.0 (1.7 to 2.3)	4.7 (3.8 to 6.3)
**Outcome type**						
preferred outcomes	8	920	198/462	85/458	2.3 (1.9 to 2.9)	4.1 (3.3 to 5.4)
lower preference outcomes	6	582	173/309	108/273	1.6 (1.4 to 1.9)	6.1 (4.1 to 12)
**Condition**						
knee osteoarthritis	5	567	127/307	58/260	2.0 (1.6 to 2.6)	5.3 (3.8 to 8.6)
other musculoskeletal	9	935	244/464	135/471	1.9 (1.6 to 2.2)	4.2 (3.3 to 5.6)

**Table 2:** Placebo contolled trials

	**Number of**		**Events/total**		
		
**Type of adverse event**	**Trials**	**Patients**	**Treatment**	**Placebo**	**RR (95% CI)**
Local adverse events	15	1734	53/949	48/785	1.0 (0.7 to 1.4)
Systemic adverse events	16	1838	33/1002	14/836	1.7 (0.96 to 2.85)
Withdrawals beacuse of adverse events	10	1225	10/697	7/528	0.9 (0.4 to 2.1)
**Active controlled trials: topical vs oral**					
	**Number of**		**Events/total**		
**Type of adverse event**	**Trials**	**Patients**	**Treatment**	**Placebo**	**RR (95% CI)**
		
Local adverse events	2	443	19/243	4/118	3.0 (1.1 to 8.5)
Systemic adverse events	3	764	82/408	87/356	0.83 (0.6 to 1.1)
Withdrawals because of adverse events	3	764	19/408	24/356	0.7 (0.4 to 1.3)

**Table 3:** Comparison of topical analgesics in chronic musculoskeletal pain

	**Number of**		**Percent success with**		
		
**Topical analgesic**	**Trials**	**Patients**	**Treatment**	**Placebo**	**NNT (95% CI)**
NSAID	14	1502	48	26	4.6 (3.8 to 5.9)
Salicylates	6	429	54	36	5.3 (3.6 to 10)
Capsaicin	3	368	38	25	8.1 (4.6 to 34)
