# Supplementary material for: Topical NSAIDs for chronic musculoskeletal pain: systematic review and meta-analysis
Source: BMC Musculoskelet Disord. 2004 Aug 19;5:28. doi: 10.1186/1471-2474-5-28 (PMC516039; doi:10.1186/1471-2474-5-28)
Supplement: Additional File 1 — Search strategy for RCTs of topical NSAIDs in chronic pain [file 1471-2474-5-28-S1.doc]

**Additional file 1: Search strategy for RCTs of topical NSAIDs in chronic pain**

#1 randomised control trial (MeSH term)

#2 random* OR blind OR double-blind OR double-masked OR masked OR trial

**#3**  #1 OR #2

#4 administration, topical (MeSH term)

#5 topical* OR cutaneous OR dermal OR transcutaneous OR transdermal OR percutaneous OR skin OR massage OR embrocation OR gel OR ointment OR aerosol OR cream OR crème OR lotion OR mouse OR foam OR liniment OR spray OR rub OR balm OR salve OR emulsion OR oil OR patch OR plaster

**#6** #4 OR #5

#7 musculoskeletal diseases (MeSH)

#8 pain* OR analgesi* OR arthrit* OR rhemat* or osteoarth* OR tend?nitis OR sciatica OR lumbago OR fibrositis

**#9** #7 OR #8

**#10** bufexamac OR bufexine OR calmaderm OR ekzemase OR dicoflenac OR solaraze OR pennsaid OR voltarol OR emugel OR voltarene OR voltarol OR optha OR voltaren OR etofenamate OR afrolate OR algesalona OR bayro OR deiron OR etofen OR flexium OR flogoprofen OR rheuma-gel OR rheumon OR traumalix OR traumon OR zenavan OR felbinac OR dolinac OR flexfree OR napageln OR target OR traxam OR fentiazac OR domureuma OR fentiazaco OR norvedan OR riscalon OR fepradinol OR dalgen OR flexidol OR cocresol OR rangozona OR reuflodol OR pinazone OR zepelin OR flufenamic OR dignodolin OR rheuma OR lindofluid OR sastridex OR lunoxaprofen OR priaxim OR flubiprofen OR fenomel OR ocufen OR ocuflur OR “Trans Act LAT” OR tulip OR ibuprofen OR cuprofen OR “deep relief” OR fenbid OR ibu-cream OR ibugel OR ibuleve OR ibumousse OR ibuspray OR “nurofen gel” OR proflex OR motrin OR advil OR radian OR ralgex OR ibutop OR indomethacin OR indocin OR indospray OR isonixin OR nixyn OR ketoprofen OR tiloket OR oruvail OR powergel OR solpaflex OR ketorolac OR acular OR trometamol OR meclofenamic OR naproxen OR naprosyn OR niflumic OR actol OR flunir OR niflactol topico OR niflugel OR nifluril OR oxyphenbutazone OR californit OR diflamil OR otone OR tanderil OR piketoprofen OR calmatel OR triparsean OR piroxicam OR feldene OR pranoprofen OR oftalar OR pranox OR suxibuzone OR danilon OR flamilon OR ufenamate OR fenazol

**#11 #3 AND #6 AND #9 AND #10**

#12 vagin* OR cervix OR cervical OR tubal OR ligation OR thrombophlebitis OR rectal OR suppository OR intra-articular OR intra-muscular OR intra-venous OR morphine OR warfarin OR nebuliz* OR PCA OR foetal OR endoscop* OR laporoscop*

**#13 #11 NOT #12**
