# Supplementary material for: Topical NSAIDs for chronic musculoskeletal pain: systematic review and meta-analysis
Source: BMC Musculoskelet Disord. 2004 Aug 19;5:28. doi: 10.1186/1471-2474-5-28 (PMC516039; doi:10.1186/1471-2474-5-28)
Supplement: Additional File 3 — QUOROM flow diagram [file 1471-2474-5-28-S3.doc]

# QUOROM Flow Diagram

60 potential studies identified

25 studies satisfied inclusion criteria and contributed data

18 placebo-controlled trials available for analysis

14 trials analysed for efficacy

4 trials had no useable data

8 trials analysed for adverse events and/or withdrawals

2 trials had no data

23 studies failed to meet inclusion criteria

12 studies had no useable data

10 active-controlled trials available for analysis

18 trials analysed for adverse events and/or withdrawals

3 trials analysed for comparison of oral and topical efficacy

7 trials compared various different topicals, doses or formulations
